# Effects of cortico-cortical paired associative stimulation based on multisensory integration to brain network connectivity in stroke patients: study protocol for a randomized doubled blind clinical trial

**DOI:** 10.1186/s12883-023-03218-2

**Published:** 2023-04-28

**Authors:** Jun-Peng Zhang, Xiang-Xin Xing, Mou-Xiong Zheng, Jia-Jia Wu, Xin Xue, Yu-Lin Li, Xu-Yun Hua, Shu-Jie Ma, Jian-Guang Xu

**Affiliations:** 1 School of Rehabilitation Science, Shanghai University of Traditional Chinese Medicine, No.1200 Cailun Road, Shanghai, China; 2 Department of Rehabilitation Medicine, Yueyang Hospital of Integrated Traditional Chinese and Western Medicine, Shanghai University of Traditional Chinese Medicine, Shanghai, China; 3 Department of Traumatology and Orthopedics, Yueyang Hospital of Integrated Traditional Chinese and Western Medicine, Shanghai University of Traditional Chinese Medicine, Shanghai, China; 4 Engineering Research Center of Traditional Chinese Medicine Intelligent Rehabilitation, Ministry of Education, Shanghai, China; 5 Rehabilitation Department of Traditional Chinese Medicine, The Second Rehabilitation Hospital of Shanghai, No. 25, Lane 860, Changjiang Road, Baoshan District, Shanghai, 200441 China

**Keywords:** Stroke, Transcranial magnetic stimulation, Spike-timing dependent plasticity, Cortico-cortical paired-association stimulation, Primary visual cortex, Primary motor cortex, Multisensory integration

## Abstract

**Introduction:**

: Brain has a spontaneous recovery after stroke, reflecting the plasticity of the brain. Currently, TMS is used for studies of single-target brain region modulation, which lacks consideration of brain networks and functional connectivity. Cortico-cortical paired associative stimulation (ccPAS) promotes recovery of motor function. Multisensory effects in primary visual cortex(V1) directly influence behavior and perception, which facilitate motor functional recovery in stroke patients. Therefore, in this study, dual-targeted precise stimulation of V1 and primary motor cortex(M1) on the affected hemisphere of stroke patients will be used for cortical visuomotor multisensory integration to improve motor function.

**Method:**

This study is a randomized, double-blind controlled clinical trial over a 14-week period. 69 stroke subjects will be enrolled and divided into sham stimulation group, ccPAS low frequency group, and ccPAS high frequency group. All groups will receive conventional rehabilitation. The intervention lasted for two weeks, five times a week. Assessments will be performed before the intervention, at the end of the intervention, and followed up at 6 and 14 weeks. The primary assessment indicator is the ‘Fugl-Meyer Assessment of the Upper Extremity ’, secondary outcomes were ‘The line bisection test’, ‘Modified Taylor Complex Figure’, ‘NIHSS’ and neuroimaging assessments. All adverse events will be recorded.

**Discussion:**

Currently, ccPAS is used for the modulation of neural circuits. Based on spike-timing dependent plasticity theory, we can precisely intervene in the connections between different cortices to promote the recovery of functional connectivity on damaged brain networks after stroke. We hope to achieve the modulation of cortical visuomotor interaction by combining ccPAS with the concept of multisensory integration. We will further analyze the correlation between analyzing visual and motor circuits and explore the alteration of neuroplasticity by the interactions between different brain networks. This study will provide us with a new clinical treatment strategy to achieve precise rehabilitation for patient with motor dysfunction after stroke.

**Trial registration:**

This trial was registered in the Chinese Clinical Trial Registry with code ChiCTR2300067422 and was approved on January 16, 2023.

## Introduction

Stroke accounts for 10% of global deaths, and the lifetime risk of stroke is estimated to be 24.9% for people over the age of 25 years worldwide. This means that one in four people will have a stroke, with the risk of ischemic stroke being higher than that of hemorrhagic stroke [1–3].

Stroke is the leading cause of death in China, and its burden has been increasing over the past three decades. There is a gradient of stroke incidence from north to south, with the highest incidence of stroke in northern and central China [1, 4]. The primary consequence of stroke for patients is the remaining motor dysfunction, which impedes their capacity to relearn daily living activities and work-related tasks [5, 6].

Clinically, neurorehabilitation therapies have been shown to improve the prognosis of stroke patients, but functional recovery can still be inadequate. Structural changes in the brain resulting from the stroke can severely impact patients’ prognosis and ability to recover functionally. [7]. Focal neurological deficits resulting from structural changes in the brain are associated with imbalanced tissue function and disordered neurophysiological responses in the affected primary cortex [5]. Studies have shown that neurological deficits may arise from alterations in both local and distant white matter tracts, as well as from disrupted neural interactions between widely distributed networks [8]. Previous studies have analyzed functional Magnetic Resonance Imaging (fMRI) data from thousands of healthy individuals and constructed a human brain connectome [9]. These studies have revealed that different symptoms can be caused by lesions in the same brain region, and conversely, the same symptoms can be caused by injuries in different locations. Briefly, specific compensatory patterns of structure and function in neural network interactions are associated with central plasticity and clinical improvement [10]. The analysis of brain structural or functional networks can help to identify the specific networks that different brain regions belong to, which can inform the development of precise treatments for stroke patients.

Recent developments in brain assessment methods and statistical techniques have led to increased research into the structural and functional effects of stroke on the brain [11]. The normal functioning of the brain is dependent on a dynamic equilibrium between the specialized functions of local brain regions, the transmission of information through neural circuits, and the integration of information across large-scale functional neural networks [12, 13]. Previous research has demonstrated that focal deficits can impact the entire brain and its network properties, leading to the idea that stroke can be seen as a disruption of brain networks [8]. Therefore, it is worth exploring whether brain dysfunction can be restored by regulating the networks.

The optimal control theory of movement requires the integration of multiple sensory systems, such as vision and proprioception. The effective functioning of multiple sensory systems is crucial for maintaining motor function. Relevant studies have suggested that visual compensation can facilitate the recovery of motor function in patients with stroke. [14]. Additional studies have demonstrated that peripheral co-excitation can spread centrally within auditory and somatosensory networks, while also eliciting brain responses in other networks involved in multisensory integration [15]. The brain has a powerful ability to automatically and simultaneously process and integrate sensory information. Integrating information from multiple sensory modalities contributes to the recovery of stroke patients in motor relearning by detecting, discriminating, and recognizing sensory stimulation [16]. While, the brain areas related to multisensory processing and those related to motor control are located in separate networks.

Previous studies have demonstrated that repetitive transcranial magnetic stimulation (rTMS) targeting specific cortical areas can effectively modify neural circuits, even the networks [17]. Transcranial magnetic stimulation (TMS) is a method of delivering electrical stimulation through the scalp [10]. Single-pulse TMS is always used to assess the activity threshold of the brain, whereas rTMS is used to induce changes in brain activity, such as affecting blood flow and metabolism in some brain regions, modulating neurotransmitter expression in the brain, and preventing long-term damage to brain tissue from delayed neurological death caused by ischemia. [18–21]. In the last few years, researchers have made considerable progress in using TMS for stroke research [22, 23]. The effect of rTMS on the excitability of networks is considered a promising strategy for the rehabilitation of stroke patients [24, 25].

The mechanism of rTMS is associated with information processing in the nervous system, which includes synaptic excitation, synaptic inhibition, and synaptic plasticity of neurons [23, 26]. The neuroplasticity with synaptic changes based on long-term potentiation and depression plays a crucial role in functional recovery after stroke [27, 28]. As an intriguing therapeutic stimulation modality of TMS, cortico-cortical paired associative stimulation (ccPAS) modulates cortical plasticity by stimulating different cortices using dual coils, which allows for the direct activation of the cortico-cortical pathway connecting two areas [29, 30]. Meanwhile, it was unexpected to find that ccPAS is consistent with spike-timing dependent plasticity (STDP), a theory related to the precise timing of pre- and post-synaptic action potentials, by manipulating the interstimulus interval between the two stimulus points [31, 32]. Then, researchers began to investigate the bidirectional modulation of cortical plasticity using different ccPAS protocols [33–35]. The use of STDP theory in TMS research is gradually increasing. In one study, investigators used a ccPAS protocol to induce bidirectional cortico-cortical associative plasticity in the posterior parietal cortex (PPC)-M1 network. When PPC preceded M1 stimulation (ccPAS + 5 ms), there was a long-lasting decrease in the excitability of M1, indicating a long-term depression (LTD)-like effect. Conversely, when PPC followed M1 stimulation (ccPAS − 5 ms), there was a long-lasting increase in the excitability of M1, indicating a long-term potentiation (LTP)-like effect [34, 36].

As a result, ccPAS could be utilized to modulate the information processing between different brain networks. The application of ccPAS between different networks would help us better understand the neurophysiological substrates of complex sensorimotor interactions and multisensory integration [30].

Naturally, our aim was to use STDP-based ccPAS to investigate whether the integration of sensory and motor systems, or networks, can enhance motor function recovery in stroke patients. In this study, we selected M1 and V1 as the stimulation targets for the motor and visual networks. M1 is a crucial component of the motor network and plays a significant role in movement execution. Previous TMS studies have used M1 as a stimulation target, demonstrating that its activation can improve motor function recovery [37]. Multisensory effects in V1 can directly influence behavior and perception, which can facilitate motor functional recovery in stroke patients [38]. Additionally, researchers conducted a ccPAS study of the visual motion area (V5) and V1 [39], where they found that: (1) paired associative TMS of human V5-V1 affects the perception of motion coherence and (2) TMS plastic strengthening of reentrant V5-V1 connections enhances motion sensitivity. Physiologically, there is enough distance between the skin mapping points of V1 and M1 to allow for the placement of a dual coil, which is conducive to achieving precise stimulation in this study.

The results of the current study can be used to determine whether paired associative stimulation of V1 and M1 based on the STDP theory can effectively restore motor function in stroke patients. Furthermore, it can help to elucidate the mechanisms underlying central plasticity changes during the intervention and shed light on the impact of interactions between different brain networks on brain plasticity.

## Methods and analysis

### Study design

This study is a double-blind randomized controlled trial, and the protocol is registered with the Chinese Clinical Trials Registry. A total of 69 participants will be divided 1:1:1 into three groups. The control group will receive conventional treatment plus sham ccPAS, the ccPAS low-frequency group received conventional treatment plus 0.2 HZ of ccPAS, and the ccPAS high-frequency group will receive conventional treatment plus 5 HZ of ccPAS.

**Fig. 1:**
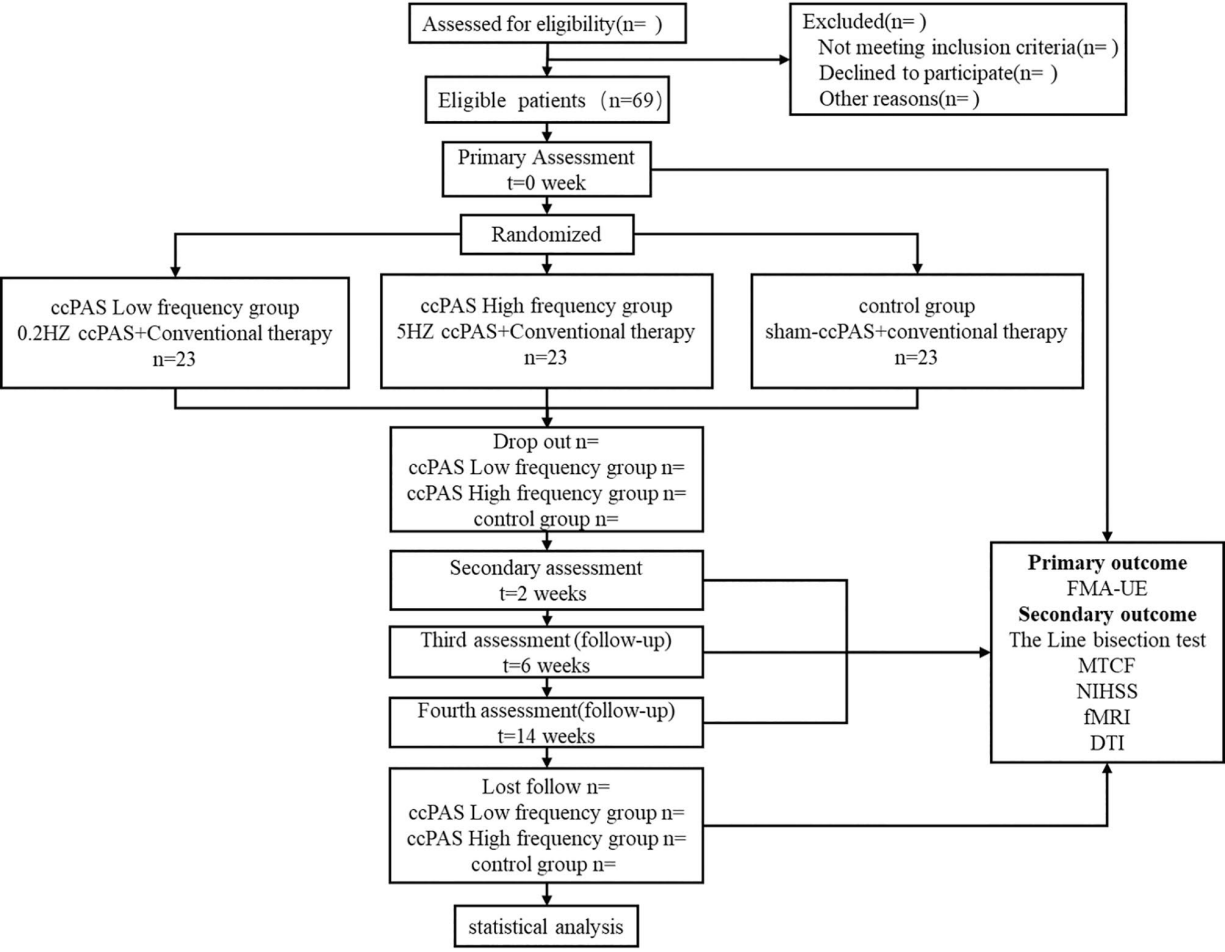
Flow diagram of study design. ccPAS, Cortical-cortical paired associative stimulation; FMA-UE, Fugl-meyer assessment of upper extremity; MTCF, Modified Taylor Complex Figure; NIHSS, National Institutes of Health Stroke Scale; fMRI, functional Magnetic Resonance Imaging; DTI, Diffusion tensor imaging

Each group will receive the intervention five times per week for two weeks. The researcher will follow up with subjects in week six and week fourteen respectively (as shown in Fig. 1 and Table 1).T The primary outcome measure will be the ‘Fugl-Meyer Assessment of the Upper Extremity (FMA-UE)’, secondary outcomes will include ‘The line bisection test’, ‘Modified Taylor Complex Figure (MTCF)’, ‘NIH stroke scale (NIHSS)’, and’ functional Magnetic Resonance Imaging(fMRI)’ as well as ‘Diffusion tensor imaging (DTI)’.

**Table 1:** Participant timeline

Activity			Study Stuff		Study Period						
				Prestudy	0 week	1 week	2 weeks	2 weeks end	6 weeks end	10 weeks end	
Preparation	Sample size calculation		Investigator A	X							
	Eligibility screen		Investigator B	X		X	X	X	X	X	
	Medical information		Investigator B	X							
	Informed consent		Investigator B	X							
	Random allocation		Investigator B	X							
Intervention	ccPAS Low frequency Group	Conventional therapy	Doctor B + Therapist			X	X				
		0.2 HZ ccPAS	Investigator C			X	X				
	ccPAS High frequency Group	Conventional therapy	Doctor B + Therapist			X	X				
		5 HZ ccPAS	Investigator C			X	X				
	control Group	Conventional therapy	Doctor B + Therapist			X	X				
		sham-ccPAS	Investigator C			X	X				
Assessment	Primary outcome	FMA-UE	Assessor A		X			X	X	X	
	Secondary outcomes	The Line bisection test	Assessor B								
		MTCF	Assessor B		X			X	X	X	
		NIHSS	Assessor B		X			X	X	X	
		fMRI	Imaging doctor		X			X	X	X	
		DTI	Imaging doctor		X			X	X	X	
Statistical analysis			Investigator A					X		X	

ccPAS, Cortical-cortical paired associative stimulation; FMA-UE, Fugl-meyer assessment of upper extremity; MTCF, Modified Taylor Complex Figure; NIHSS, National Institutes of Health Stroke Scale; fMRI, functional Magnetic Resonance Imaging; DTI, Diffusion tensor imaging.

### Study setting

The study is supported by the Department of Rehabilitation Medicine, Yueyang Hospital, Shanghai University of Traditional Chinese Medicine. All interventions will be conducted in the Department of Rehabilitation Medicine, Yueyang Hospital, Shanghai University of Traditional Chinese Medicine.

### Recruitment and sample selection

Subjects will be recruited by the Department of Rehabilitation Medicine, Yueyang Hospital, Shanghai University of Traditional Chinese Medicine,based on strict inclusion and exclusion criteria. Those who meet the criteria will be asked to sign an informed consent form.

### Inclusion criteria

1. Stroke (Infarction/Ischemia) [40];
2. Age ≥ 40 years and ≤ 70 years, regardless of sex [41];
3. Time of onset ≥ three months and ≤ 1 year;
4. right-handedness;
5. normal cognitive function (MMSE above the threshold corresponding to different cultural levels);
6. Informed consent;

### Exclusion criteria [42]

1. Contraindicated implants for MRI and TMS in vitro and in vivo;
2. History of epilepsy, psychiatric disorders, or other serious cognitive or emotional disorders;
3. History of medications that affect the central nervous system;
4. Pregnant or lactating women;

### Dropout criteria

1. Withdrawal from the study;
2. Incomplete implementation of the treatment plan;
3. Other treatment access;
4. Blinding failure;

### Discontinuation criteria

If a patient develops other diseases or experiences serious adverse reactions during treatment, the trial will be discontinued at the mutual decision of the subject and the investigator.

### Sample size calculation

In this study, the sample size was determined using a multiple group mean comparison method. The sample calculation formula is $n = {\varphi ^2}\left( {\sum {S_i^2/g} } \right)/\left[ {{{\sum {\left( {{{\bar X}_t} - \bar X} \right)} }^2}/\left( {g - 1} \right)} \right]$

The number of groups(g) is three, and the number of participants in the control and ccPAS low-frequency groups and the ccPAS high-frequency group was 1:1:1. α is the probability of rejecting a true null hypothesis, α is set to 0.025. β is set to 0.1, power(1-β) is the probability of rejecting a false null hypothesis. K is the group means multiplier; k is 1. The value of φ is obtained by checking the table according to α, β, v1, v2.According to previous studies, the three group means are set to 8.11 6.87 17.62, the within group standard deviation (σ) is 8.83 [43]. Using PASS 15 analysis, we get that the total sample size is 54 and the sample size of each group is 18. Considering the 20% dropout rate, the total sample size of the study should be set to 69 and the sample size of each group is 23.

### Randomization and allocation concealment

A random number table will be generated by SPSS 25.0 to create random assignment cards. These cards will then be placed in opaque envelopes with envelope serial numbers connected to the card serial numbers. Participants will be randomly assigned codes based on the order in which they are seen. The grouping specified by the card inside the envelope will determine which group each participant will be assigned to, with each group consisting of 23 participants: a control group, a ccPAS low-frequency group, and a ccPAS high-frequency group. The grouping information will be kept confidential from the patients, their families, therapists, evaluators, and statistical analysts. Patient treatment information will be sent to the researcher one day prior to the treatment.

### Blinding

Each participant will sign a written confidentiality agreement to ensure that information is not shared with others. Randomization of patients will be done remotely by a person who will not be involved in the rest of the trial process. Patients who meet the inclusion criteria will undergo a first basic assessment by a person dedicated to the assessment after signing the informed consent form.

Routine rehabilitation will be done by a physical therapist, an occupational therapist, and a speech therapist in the rehabilitation medicine department. ccPAS treatment will be done by a dedicated researcher, following the given parameters.

The two-week post-intervention assessment will be completed by a second assessor. Follow-up will be done by a third assessor. The data from all assessments will be analyzed by a dedicated data analyst, who will only be provided with the data results for each group without knowing the specific group allocation.

### Intervention

Subjects will receive regular rehabilitation, including physiotherapy, occupational therapy, and speech and language therapy. Brain areas will be localized using the LOCALITE TMS Navigator system, which will rely on the patient’s magnetic resonance structural images to localize the M1 and V1 [44]. Patients will receive ccPAS treatment with a MagTD magnetic field stimulator from Wuhan Iredell Medical Equipment New Technology Co.

### ccPAS low frequency group

The affected side of the brain will receive dual-target transcranial magnetic stimulation with V1 and M1 as the target sites. The first coil will be placed above V1, tangent to the scalp, at a 45-degree angle to the midline; the second coil will be placed at M1, tangent to the scalp, also at a 45-degree angle to the midline. The stimulation of V1 will occur 18ms before that of M1 [45–47]. The daily ccPAS treatment protocol will consist of 180 pairs of transcranial magnetic stimulation pulses at a frequency of 0.2 Hz for 15 min [48]. The patients will receive the treatment while comfortably seated with relaxed extremities. The stimulation intensity will be set at 120% AMT, which is the minimum machine output intensity that elicits five MEP (motor evoked potential) amplitudes above 100 microvolts during mild contraction of the target muscle for 10 stimulations [49, 50]. MEP was recorded by stimulating M1 to induce contraction of the first dorsal interosseous(FDI) muscle.

### ccPAS high frequency group

The daily ccPAS treatment will consist of 180 pairs of transcranial magnetic stimulation pulses at a frequency of 5 Hz, separated into five segments of 30 pairs of pulses each, with 30 s intervals between segments (3.6 min in total) [30, 51]. Other settings will be the same as the ccPAS Low frequency group.

### Control group

The sham stimulation will be achieved by a non-functional sham coil with the same external weight and sound as the real coil, and the rest of the parameters will be performed in the same way as in the ccPAS Low frequency group.

### Assessment

The patient’s basic information will be obtained from the hospital visit record. The patient’s assessment arrangements are shown in Table 1.

### Fugl-Meyer assessment of the upper extremity (FMA-UE)

The Fugl-Meyer Assessment (FMA) is a widely used method for evaluating sensorimotor impairment in stroke patients and is commonly employed in the clinical assessment of motor function [52]. The Fugl-Meyer Assessment scores have been tested repeatedly and have shown good consistency, responsiveness, and accuracy. The FMA-UE contains 33 assessment items with a total score of 66. The testers can rate the severity of the subject’s motor impairment based on their final score.

### The line bisection test

The patients will be asked to estimate and indicate the midpoint of a horizontal line. It is expected that patients with left neglect will choose a midpoint to the right of true center. Three horizontal, 8-inch black lines, 1-mm thick, will be presented to each patient in a staircase fashion across the page. The extent of each line will be clearly indicated to the patient, who will then be instructed to mark the center.

### Modified taylor complex figure (MTCF)

The Rey-Osterrieth Complex Figure (ROCF) is commonly used in neuropsychology to assess visuospatial abilities in children and adults. The Taylor Complex Figure (TCF) was developed as an alternate form for the ROCF and has been shown in numerous studies since 1990 to be easier to learn and remember than the ROCF. The Modified Taylor Complex Figure (MTCF) has also been shown in several studies to produce comparable total scores, completion time, and validity coefficients to the ROCF [53].

### National institutes of health stroke scale (NIHSS)

The NIHSS is a 15-item impairment scale, intended to evaluate neurologic outcome and degree of recovery for patients with stroke [54]. The scale assesses level of consciousness, extraocular movements, visual fields, facial muscle function, extremity strength, sensory function, coordination (ataxia), language (aphasia), speech (dysarthria), and hemi-inattention (neglect). The NIHSS was designed to assess differences in interventions in clinical trials.

### Functional magnetic resonance imaging (fMRI)

A 3.0T United Imaging uMR770 MRI system (United Imaging, Shanghai, China) with a standard cranial 32-channel coil will be used. Resting-state fMRI will be acquired using an echo-planar imaging (EPI_BOLD) sequence, with the following scan parameters: TR)/ TE : 3000/30 ms, flip angle (FA): 90°, field of view (FOV) : 220 × 220 mm, matrix size : 64 × 64, slice thickness : 3 mm, slices : 43, and a total of 240 time points were acquired with a scanning time of 12 min.

### Diffusion tensor imaging (DTI)

T1-weighted magnetization-prepared rapid acquisition will be performed with the following parameters: repetition time/inversion time/echo time = 1900/900/2.93 milliseconds, flip angle = 9°, FOV = 256 × 256 mm^2^, section thickness = 1.0 mm sagittal acquisition, acquisition matrix = 256 × 256, and number of averages = 1. Diffusion-weighted images were acquired based on a single shot spin EPI in the axial plane: repetition time/echo time = 10,000/89, flip angle 90, slice thickness 2.0 mm, in-plane resolution 1.875 mm, and 60 noncolinear directions (b = 0, 1000 s/mm^2^).

### Data management

All participants who have provided their informed consent and the results of each assessment will be recorded in both physical and electronic formats. The physical data will be stored by the assessor, who will replace the participants’ real names with their assigned numbers, and create a digital copy of the results for the data analysts. The entire file will be encrypted, and only the data analysts and assessors will have access to it for data verification, to ensure the security and reliability of the data. The Clinical Research Management Center of Yueyang Hospital of Integrated Traditional Chinese and Western Medicine (Shanghai University of Traditional Chinese Medicine) is responsible for supervising the experimental data, which will be stored for three years after the study is completed before being erased.

### Statistical analysis

#### Behavior statistical analysis

IBM SPSS Statistics 25.0 will be used to analyze the measurement data for each of the three groups at four time points. Measures will be tested for normality, and those meeting the normal distribution will be expressed as mean ± standard deviation (x ± s). Within-group comparisons, post-intervention and two follow-up visits will be compared with baseline (first assessment) using ANOVA with repeated measures data, and then two-by-two comparisons (post-hoc) will be performed to observe differences at each time point if the overall difference was statistically significant. One-way ANOVA will be used for comparison between groups, and further two-by-two comparisons were made. Measures that do not pass the normality test will be expressed as marginal, maximum, and minimum values. Non-normally distributed measures will be compared by Friedman test for a group of own pre-post comparisons and Kruska-Walls H test for between-group comparisons. The level of unity significance will be set at P = 0.05, and P < 0.05 is statistically significant.

#### fMRI statistical analysis

Preprocessing will be performed using the Statistical Parametric Mapping (SPM) package based on the MATLAB 2013b platform (The Mathworks, Inc, Natick, US).Through format conversion, slice timing, spatial normalization, spatial smoothing, head movement correction realigns (estimate & reslice), origin correction, linear trend detrend elimination, regression covariates, and filtering filter will be used to achieve noise reduction, spatial alignment, and anatomical position correspondence.

To verify whether the functional connectivity between primary visual cortex and primary motor brain regions in the brain will change after ccPAS treatment, bilateral primary motor cortex and primary visual cortex located in the cortex will be selected as ROIs based on the Montreal AAL90 template, and the correlation between brain regions will be analyzed using ROI-level functional connectivity using REST software.

#### DTI statistical analysis

DTI processing will require the use of the FMRIB Software Library v6.0 (FSL), which was created by the Analysis Group at FMRIB, Oxford, UK. FSL is a comprehensive library of analysis tools specifically designed for FMRI, MRI, and DTI brain imaging data.

On a Linux system, we will use code to complete the pre-processing part of the data, including data quality checks, data format conversion, head movement and eddy current correction, acquisition of brain mask, and tensor calculation. The ROI-based DTI analysis method will be adopted to complete the image alignment, extract ROI from the atlas, and calculate the DTI index of ROI. Deterministic fiber tracking will then be used to extract fibers from the primary visual cortex and primary motor cortex, and the corresponding indexes will be calculated. The correlation of the brain regions will be reflected by calculating the fiber tract connections between the primary visual cortex and primary motor cortex on the healthy and affected side.

## Discussion

Numerous animal and clinical trials have shown a significant trend towards structural and functional remodeling of the brain following a stroke. In particular, it is crucial to reconstruct, repair, or enhance the structural or functional connections between damaged brain regions to promote recovery of function after a stroke, with a focus on motor circuits [24, 55].

rTMS is a non-invasive treatment that uses magnetic fields generated by coils passing through the scalp to induce changes in cortical excitability [56]. It is an important tool for human brain intervention and research [57]. Previous studies have found that rTMS can induce prominent plasticity changes in the brain of post-stroke patients and modulate the recovery and reconstruction of brain function, providing a new model for the treatment of neurological and psychiatric disorders [58]. According to Donald Olding Hebb’s theory, the repeated synchronized activity of pre- and postsynaptic neurons strengthens synaptic connections, resulting in post-synaptic cell excitatory LTP, which has fast persistence, association, correlation, and input specificity. This is the basis of using TMS to modulate the brain [59, 60]. Furthermore, STDP induces LTP/LTD effects by modulating the pre- and postsynaptic excitatory sequence and stimulus timing [48, 61].

Earlier studies have indicated that ccPAS can induce changes in the human brain’s left and right motor cortex and modulate interhemispheric inhibition based on the Hebbian theory. These findings provide a theoretical basis for the plasticity of ccPAS-induced connectivity between cortical target areas, suggesting that the effectiveness of ccPAS in inducing sustained changes in excitability is dependent on the precise timing of the stimulus pair. Additionally, there have been studies on ccPAS involving the ventral premotor cortex (PMv) and M1, SMA and M1, and PPC and M1, which have effectively enhanced connectivity between relevant brain regions [33, 34, 45, 62].

Previous research has demonstrated that the primary visual cortex plays a role in multisensory processing during the early stages of stimulation. Furthermore, it has been shown that multisensory effects within this region directly impact behavior and perception, which can aid in motor function recovery [38]. By directly intervening with ccPAS in both V1 and M1 to achieve multisensory integration in the brain, we can further clarify the correlation between connectivity among brain regions and verify the effects of interactions between different brain networks on brain plasticity.

It is expected that the combination of ccPAS and the concept of multisensory integration will be effective in promoting the recovery of motor function, enhancing the functional connectivity of damaged motor neural circuits after stroke, and providing a new therapeutic option for chronic post-stroke rehabilitation.

## Data Availability

Not applicable.

## Abbreviations

ccPAS: Cortico-cortical paired associative stimulation
DTI: Diffusion tensor imaging
FMA-UE: Fugl-Meyer Assessment of the Upper Extremity
fMRI: Functional Magnetic Resonance Imaging
FSL: FMRIB Software Library
LTD: Long-term depression
LTP: Long-term potentiation
M1: Primary motor cortex
MMSE: Mini-mental State Examination
MTCF: Modified Taylor Complex Figure
NIHSS: NIH stroke scale
PMv: Ventral premotor cortex
PPC: Posterior parietal cortex
ROCF: Rey-Osterrieth Complex Figure
ROI: Region of interest
rTMS: Repetitive transcranial magnetic stimulation
SPM: Statistical Parametric Mapping
STDP: Spike-timing dependent plasticity
TCF: Taylor Complex Figure
TMS: Transcranial magnetic stimulation
V1: Primary visual cortex
